# Supplementary material for: Accurate trajectory inference in time-series spatial transcriptomics with structurally-constrained optimal transport
Source: bioRxiv. 2025 Mar 19:2025.03.19.644194. Preprint. [Version 1] doi: 10.1101/2025.03.19.644194 (PMC11957147; doi:10.1101/2025.03.19.644194)
Supplement: Supplement 1 [file media-1.pdf]

Supplemental Materials

Supplemental Table 1: Clustering resolution parameters for organogenesis data

Dataset	Leiden resolution parameter
Heart E14.5	0.25
Heart E16.5	0.35
Kidney E15.5	0.45
Kidney E16.5	0.85
Lung E14.5	0.65
Lung E15.5	0.3
Lung Epithelium E14.5	0.45
Lung Epithelium E15.5	0.45

Supplemental Table 2: Clustering resolution parameters for ovulation data

Dataset	Leiden resolution parameter
0h Ovary	1.5
4h Ovary	1.5
0h Immature follicles	0.15
4h Immature follicles	0.15

Supplemental Table 3: Differentially expressed genes in maturing preantral follicles (SOCS map)

Gene Name	Fold-change	Log_{10}(-q-value)
Rspo1	-1.754421	4.998933
Wnt6	-1.457247	8.737851
Pcsk6	-1.290005	10.60675
Adamts1	1.32581	1.306304
Rragd	1.33831	5.012801
Nap1l5	1.35748	2.877374
Nr4a1	1.4304	4.481518
Prlr	1.43665	1.404911
Fzd1	1.47788	1.404911
Star	1.4885	3.440808
Rgcc	1.49512	4.862264
Fdx1	1.52156	6.683389

Mgarp	1.53592	4.742552
Gm2a	1.60467	10.86491
Runx1	1.61162	2.155102
Coch	1.63063	5.386973
Inhba	1.65062	6.07295
Apoe	1.66863	14.58734
C1s1	1.74246	9.269438
Runx2	1.74501	2.165012
Krt8	1.89647	7.621121
Vim	1.96513	1.851203
Mro	2.09918	14.58734
Sphk1	2.15805	4.017297
H2-Ab1	2.29504	1.75097
Rtp4	2.39654	1.891041
Junb	2.93652	7.374893
Dusp4	3.66005	6.030907

Supplemental Table 4: Differentially expressed genes in maturing preantral follicles (W-OT map)

Gene name	Fold-change	Log_{10}(-q-value)
Pcsk6	-1.444813	30
Wnt6	-1.422421	7.497008
Rspo1	-1.408379	1.92808
Vcan	-1.339587	1.928102
Fkbp6	-1.323484	1.332118
Rasd1	-1.317828	13.69269
Kitl	-1.262368	4.842375
Aldh1a1	1.31597	6.062563
Cyp17a1	1.3202	1.942498
Cyp11a1	1.34254	2.293042
Fdx1	1.34435	3.602534
Gm2a	1.37122	5.126492
Adamts1	1.39685	1.783297

Apoe	1.40973	6.737819
Inhba	1.47267	3.979453
Nr4a1	1.51851	6.020553
C1s1	1.5338	5.905647
Runx2	1.55372	1.55525
Egr1	1.56617	2.68166
Mro	1.56701	5.902857
Runx1	1.58271	2.05766
Coch	1.59561	5.081843
Mgarp	1.68814	6.772438
Star	1.74612	6.354732
Prlr	1.98017	4.042679
Sphk1	2.01921	3.624881
Dusp4	2.07076	3.2271
Junb	2.88375	7.578948

Supplemental Table 5: Differentially expressed genes in maturing preantral follicles (Moscot map)

Gene Name	Fold-change	Log_{10}(-q-value)
Fshr	-1.26863	3.136819
Aldh1a1	-1.32021	5.403561
Nap1l5	-1.32886	1.968575
Nr4a1	-1.40651	3.38821

Supplemental Table 6: Differentially expressed genes in stromal neighbors of maturing preantral follicles (SOCS map)

Gene name	Fold-change	Log_{10}(-q-value)
Prtn3	2.892106	2.770555
Inhba	1.48547	4.287642
Adamts1	1.459067	4.947472
Prlr	1.442901	3.073363
Fdx1	1.435678	5.452425
Cyp17a1	1.34844	2.730145
Star	1.337649	2.557454
C1s1	1.302854	3.578336
Inha	1.280972	2.770555

Supplemental Figures

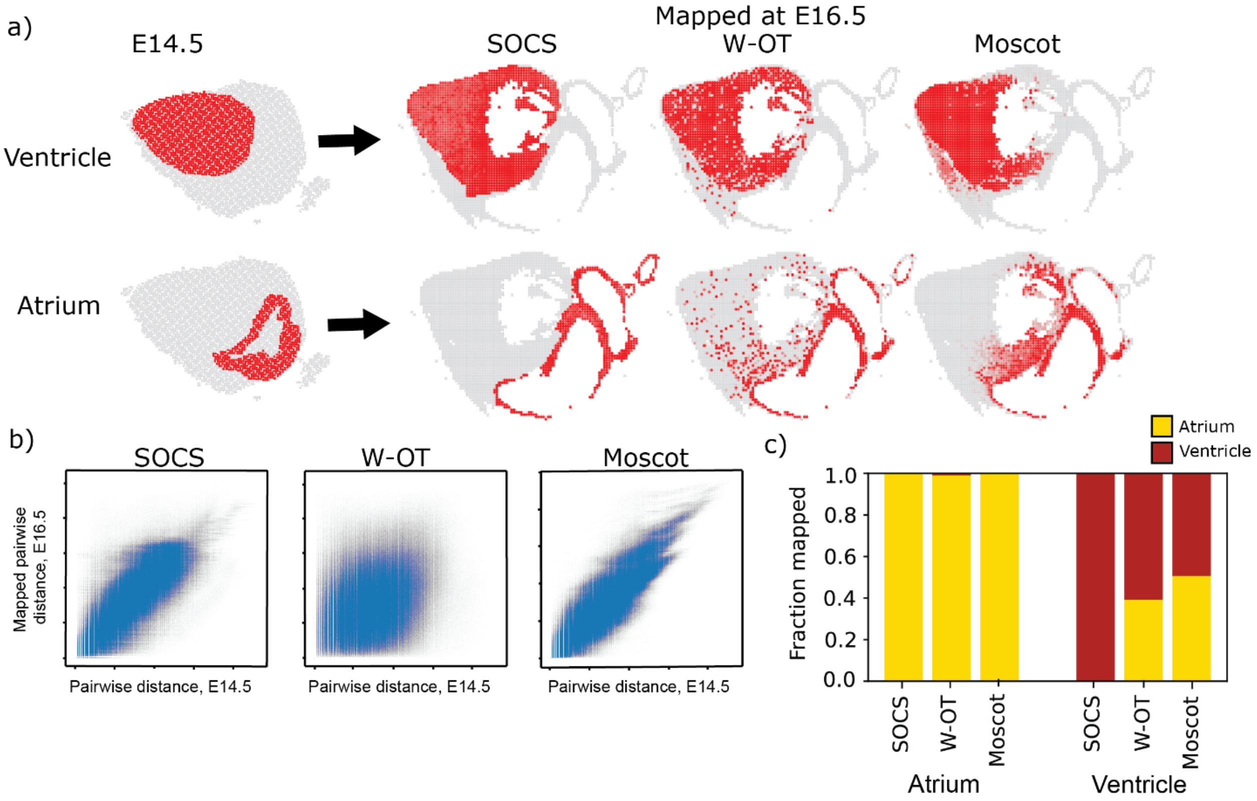

Supplemental Figure 1 SOCS analysis of time-series Stereo-seq in developing mouse heart: a) Cells from ventricle and atrium from mouse heart obtained at E14.5 mapped to E16.5 by W-OT, Moscot, and SOCS. b) Scatter plots comparing pairwise spatial distances for each cell pair at E14.5 to their mapped pairwise distances at E16.5. For SOCS, Pearson's $r = 0.71$, for W-OT, Pearson's $r = 0.27$, and for Moscot, Pearson's $r = 0.84$. c) Cell type mapping between mouse heart at E14.5 and E16.5, by mapping method.

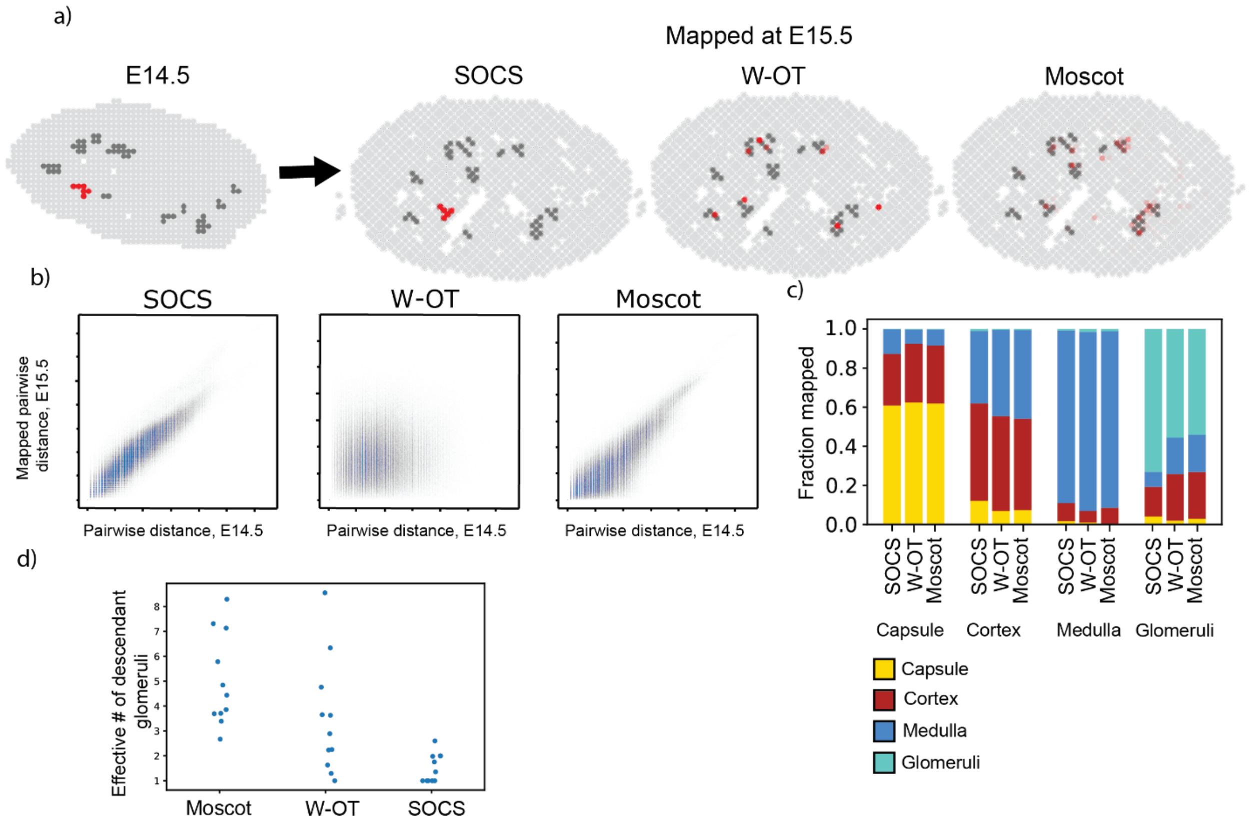

Supplemental Figure 2 SOCS analysis of time-series Stereo-seq in developing mouse kidney: a) Cells from a single glomerulus from mouse kidney obtained at E15.5 mapped to E16.5 by W-OT, Moscot, and SOCS. b) Scatter plots comparing pairwise spatial distances for each cell pair at E15.5 to their mapped pairwise distances at E16.5. For SOCS, Pearson's $r = 0.89$, for W-OT, Pearson's $r = 0.10$, and for Moscot, Pearson's $r = 0.84$ c) Cell type mapping between mouse kidney at E15.5 and E16.5, by mapping method. SOCS exhibits substantially stronger mapping from glomeruli to glomeruli than W-OT or Moscot. d) Strip plot showing effective number of descendant glomeruli at E16.5 for each glomerulus at E15.5 ($n = 12$), by mapping method. In the SOCS map, E15.5 glomeruli have a mean of 1.51±0.55 effective descendant glomeruli, compared to 3.48±2.20 in the W-OT map and 5.01±1.77 in the Moscot map.

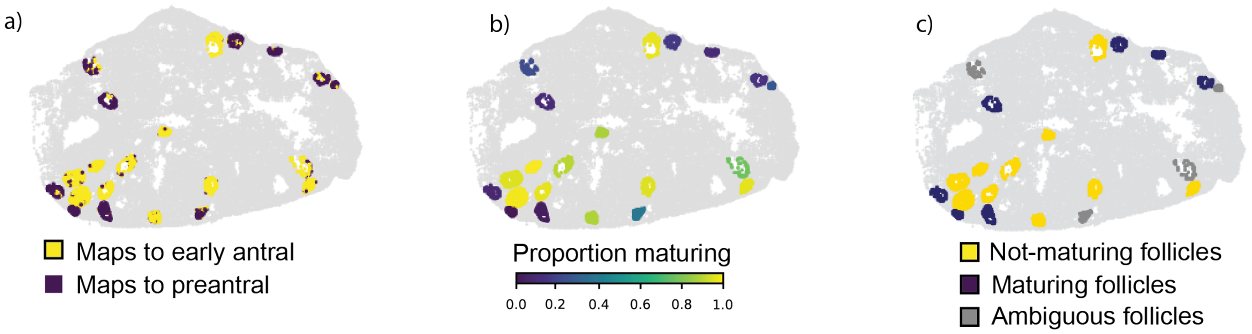

Supplemental Figure 3 Categorizing maturing follicles: a) Preantral follicle cells labeled by mapping to preantral or early antral follicle cells. b) Preantral follicles, colored by proportion of their cells mapping to early antral follicles. c) Follicles with 80% or more of cells mapping to early antral follicles labeled as “maturing,” follicles with 80% of more of cells mapping to preantral follicles labeled as “not maturing.”

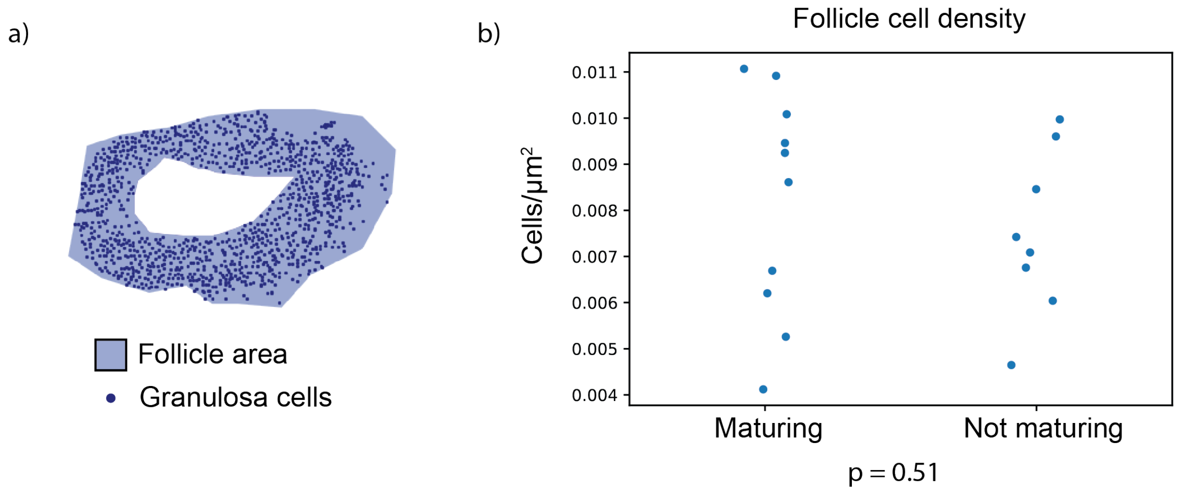

Supplemental Figure 4 Follicle cell density comparison: a) Computation of follicle density: the number of granulosa cells in the follicle is divided by the segmented follicle area. b) Comparison of cell density of maturing and not-maturing preantral follicles.

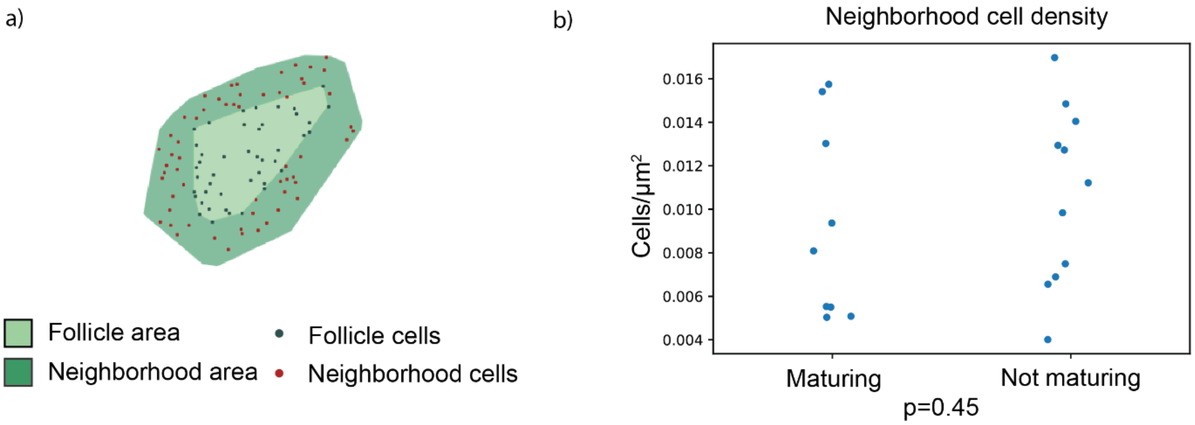

Supplemental Figure 5 Follicle environmental density: a) Computation of follicle neighborhood density: the number of cells in the follicle neighborhood is divided by the area of the neighborhood (obtained from the convex hull of the neighborhood cells). b) Comparison of neighborhood density of maturing and not-maturing preantral follicles

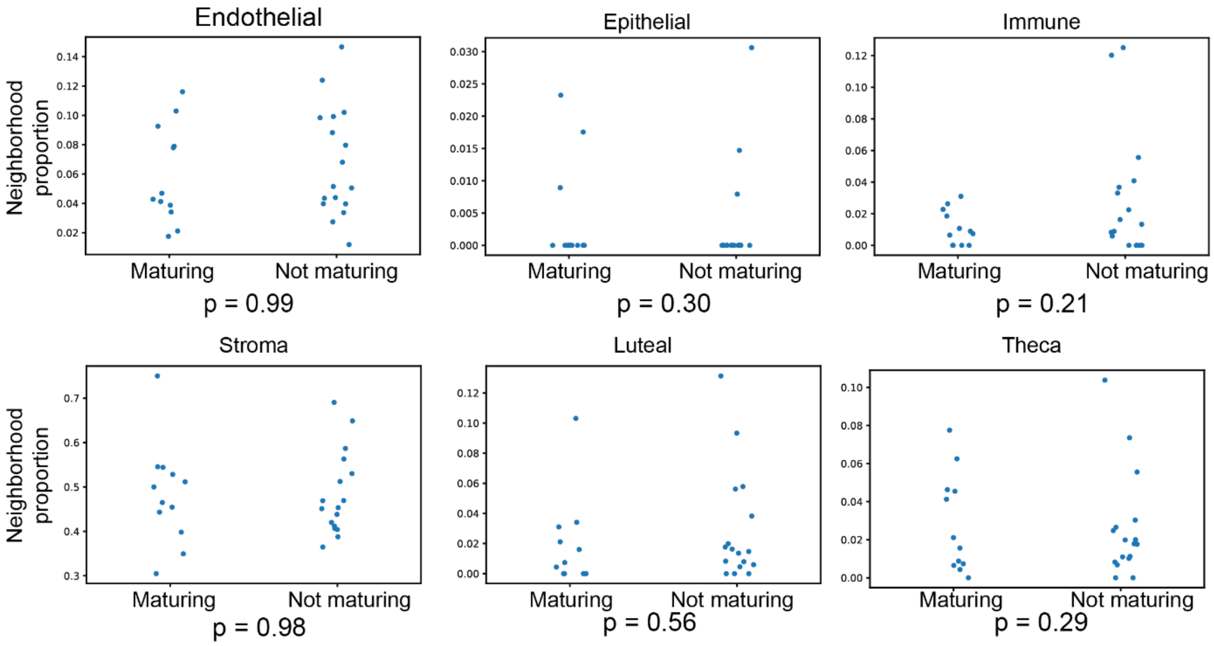

Supplemental Figure 6: For each major cell type, comparison of follicle neighborhood proportion in maturing and not-maturing preantral follicles.
